# Regression of Castration-Resistant Prostate Cancer by a Novel Compound HG122

**DOI:** 10.3389/fonc.2021.650919

**Published:** 2021-06-03

**Authors:** Xiaonan Cong, Yundong He, Haigang Wu, Dingxiang Wang, Yongrui Liu, Ting Shao, Mingyao Liu, Zhengfang Yi, Jianghua Zheng, Shihong Peng, Tao Ding

**Affiliations:** ^1^ East China Normal University and Shanghai Fengxian District Central Hospital Joint Center for Translational Medicine, Shanghai Key Laboratory of Regulatory Biology, Institute of Biomedical Sciences and School of Life Sciences, East China Normal University, Shanghai, China; ^2^ Department of Laboratory Medicine, Shanghai University of Medicine & Health Sciences Affiliated Zhoupu Hospital, Shanghai, China; ^3^ Department of Urology, Shanghai Jiao Tong University Affiliated Sixth People’s Hospital South Campus, Shanghai, China; ^4^ Southern Medical University Affiliated Fengxian Hospital, Shanghai, China

**Keywords:** HG122, castration-resistant prostate cancer, androgen receptor, cancer treatment, molecular compound

## Abstract

Prostate cancer (PCa) is a common aggressive disease worldwide which usually progresses into incurable castration-resistant prostate cancer (CRPC) in most cases after 18–24 months treatment. Androgen receptor (AR) has been considered as a crucial factor involved in CRPC and the study of AR as a potential therapeutic target in CRPC may be helpful in disease control and life-cycle management. In this study, we identified a potent small molecule compound, HG122, that suppressed CRPC cells proliferation and metastasis, and inhibited tumor growth both in subcutaneous and orthotopic tumor model. In addition, HG122 reduced the mRNA expression of PSA and TMPRSS2 which are target genes of AR, resulting in cell growth inhibition and metastasis suppression of CRPC, without affecting the expression of AR mRNA level. Mechanically, HG122 promoted AR protein degradation through the proteasome pathway impairing the AR signaling pathway. In conclusion, HG122 overcomes enzalutamide (ENZ) resistance in CRPC both *in vitro* and *in vivo*, thus suggesting HG122 is a potential candidate for the clinical prevention and treatment of CRPC.

## Introduction

191,930 cases of prostate cancer (PCa) were estimated to be diagnosed and 33,330 PCa patients were estimated to die in 2020 in the United States (1). The majority of PCa patients expresses androgen receptor (AR) at high levels and responds to androgen deprivation therapies (ADT, surgical or medical castration) (2–4), but patients usually relapse and evolves into castration resistant prostate cancer (CRPC) within 2–3 years, finally leading to death (5). Improving and optimizing therapeutic strategies for men with CRPC is one of the most challenging aspects of PCa management. Treatment options for such patients are limited, and chemotherapy provides response rates of only 6% (6–9). However, because of recent successes in the development of less toxic regimens and effective markers for tumor response, chemotherapy can be used to induce a regression in CRPC after failure of ADT (10–13). CRPC patients express AR and androgen-regulated genes at high levels, indicating that AR transcriptional activity is at least partially reactivated despite the castrate serum androgen level (14, 15). Accumulating evidences suggest that AR is a critical player in early-stage PCa as well as late-stage CRPC (16–18). Thus, identifying effective inhibitors of AR signaling that may act independently in hormonal status is of paramount interest. This strategy may reduce the morbidity and mortality of PCa patients as well as prevent the progression of early tumors to life-threatening CRPC tumor phenotypes. Thus, agents that inhibit AR signaling may be extremely useful for PCa treatment.

AR, a member of the nuclear hormone receptor superfamily (19–21), is either activated by binding of dihydrotestosterone (DHT) (22, 23) or constitutively activated. Binding to the AR ligand-binding pocket induces a conformational change of AR, and AR interacts with co-activators in a unique manner to be transferred into the nucleus. AR binds specific recognition sequences known as androgen response elements (AREs) in the promoter and enhancer regions of target genes, ultimately resulting in modulation of gene expression (24). It has always been believed that reducing serum androgen levels in prostate cancer patients by surgery or chemical methods can completely inhibit the AR signaling pathway and inhibit the progression of prostate cancer (25). While recent evidences show that in CRPC, the AR signaling pathway is still active even when serum androgen is at castrated levels. Even under castration conditions, AR can be activated through a variety of mechanisms, including: primary androgen synthesis (26), AR caused by AR overexpression, mutation or splicing (27), interaction of co-acting factors, and post-translational modification (28). These findings have led to clinical development and adoption of new therapeutic methods targeting AR signaling pathways not only for PCa but for CRPC. Thus, agents that inhibit AR signaling might be extremely useful for treating CRPC.

## Materials and Methods

### Synthesis of HG122

HG122 was screened and identified as an inhibitor of AR transcriptional activity. Its synthetic protocol was shown in **Supplementary Figure 1** and described as below. Para-anisidine (compound 1, 10 mmol) was suspended into HCl aqueous solution (1%, 50 mL) and continuously stirred in the ice bath. Then, NaNO_2_ (1.1eq, 11 mmol) in 10 mL of cooled aqueous solution was dropwise added to the para-anisidine solution and continued to be stirred for another 1 h, confirming that all the amino group of anisinde were oxidized to diazonium salt (compound 2). The 4-Nitro-o-phenylenediamine (10 mmol) was added to that and stirred in ice bath overnight. The pH of mixture was adjusted to more than 9.0 and free azo compounds were extracted using EtOAc. The organic layer was dried using Na_2_SO_4_ and the organic solvent was removed using vacuum evaporation machine to obtain dried azo compound (compound 3). This azo compound **3** was not purified and further dissolved into the 50 mL of pyridine. The 10 g of CuSO_4_ was added to the 50 mL of concentrated NH_3_·H_2_O aqueous in the ice bath. The CuSO_4_/NH_3_ solution was slowly added to the azo pyridine solution and further refluxed in the oil bath (120 degree) overnight. When reaction finished, the pyridine was removed using vacuum evaporation machine and amino-triazole product was extracted using EtOAc. This crude product (compound 4) was purified using column chromatography with EtOAc/PE as flowing phase. The amino-triazole (4, 2 mmol) was dissolved into cooled CH_2_Cl_2_ (10 mL, DCM) and 6 mmol of Et_3_N was added to this solution. The chloroacetyl chloride (1.25eq., 2.5 mmol) was dropwise added to the DCM solution and further stirred until no amino-triazole was observed in the reacting mixture. The crude product was purified using column chromatography to obtain the white powder product (compound 5). The compound synthesized in last paragraph (1 mmol) was dissolved into acetone and 2 mmol of AgNO_3_ was added. The white powder was precipitated and further removed. The final product (compound 6) in acetone solution was purified using column chromatography and further characterized using ^1^H NMR and HPLC-MS to examine the molecular structure.

### Cell Culture, Animals, and Reagents

All human PCa cell lines used in this study were purchased from ATCC. PCa cells were cultured under standard cell culture conditions at 37°C and under 5% CO2 in RPMI 1640 (Gibco, MA, USA) supplemented with 10% FBS (Biowest, Nuaille, FR) and 1% penicillin/streptomycin, unless otherwise specified. RWPE-1 was cultured in keratinocyte serum-free medium (Invitrogen, CA, USA). In addition, 22RV1 cells were transfected with pGL4 vector (Promega, WA, USA) and selected in G418 for stable 22RV1-luc cell line. Mice were obtained from National Rodent Laboratory Animal Resources, Shanghai Branch, China. All animal experimental protocols were approved by the Animal Investigation Committee of the Institute of Biomedical Sciences, East China Normal University. The AR (N20) antibody was purchased from Santa Cruz, and β-actin antibody was purchased from Sigma-Aldrich. The secondary antibody was conjugated with IRDye 680/800 (Millennium Science, VIC, AU). The compound HG122, with a purity greater than 98%, was synthesized by our lab.

### Cell Viability Assay

Human PCa cells were seeded in 96-well plates (5 × 10^3^ cells/well). After 12 h, the medium was removed, and cells were exposed to various concentrations of HG122 (0, 2.5, 5, 10, and 20 μM) for 48 h or 72 h with or without DHT (10 nM). MTS (25 μL) was added and cells were incubated at 37°C for approximately 1 h; spectrophotometric absorbance was then measured using a 96-well plate reader SpectraMax-190 (Molecular Devices, CA, USA) at 490 nm. IC50 was calculated using the GraphPad Prism 6 (GraphPad software, CA, USA). The number of viable cells was calculated relative to those in controls. AR antagonist enzalutamide and bicalutamide (BIC) were served as positive controls. The assay was repeated three times independently.

### Colony Formation Assay

For the colony formation assay, PCa cells were seeded into 6-well plates. Cells were allowed to attach to the plate surface overnight, incubated with indicated concentrations of HG122 for 1 week, and fixed with 4% paraformaldehyde for 20 min at room temperature. After staining with 0.2% crystal violet, colonies were visualized under a microscope and analyzed as the ratio of the number of colonies in treated samples to that in untreated samples. The assay was repeated three times independently.

### Transwell Invasion Assay

It was executed using a Boyden chamber (Falcon, NJ, USA) with 8.0-μm pore size in 24-well plates. The top chambers were coated with 20 μL Matrigel (BD Biosciences, NJ, USA) and incubated for 30 min at 37°C for polymerization. Then, 1 × 10^4^ cells in serum-free medium with various concentrations of HG122 were seeded into the top chambers. The bottom chambers were filled with complete medium containing 10 nM DHT and various concentrations of HG122. After a 12-h incubation at 37°C, non-invaded cells were scraped with a cotton swab, and invaded cells were fixed with 4% paraformaldehyde and stained with 0.1% crystal violet. Images were obtained, and cell numbers were quantified by manual counting. Three independent experiments were performed.

### Dual Luciferase Screening Assay and Plasmids

PCa cells were transfected with MMTV-luc and Renilla-luc (Promega, WA, USA) using lipofectamine 2000 (Invitrogen, CA, USA) according to the manufacturer’s instructions. After transfection for 24 h, the transfected cells were treated with or without DHT (Sigma-Aldrich, MO, USA) and HG122 for 12 h. Renilla and firefly activities were determined by luminometry using the Dual-Luciferase Reporter Assay System (Promega, WA, USA), and their ratio was calculated. Results were expressed as the ratio of firefly to renilla luciferase activities (n = 3).

### Quantitative Real-Time PCR

LNCaP and 22RV1 cells were cultured in 5% charcoal dextran-treated FBS for 5 days and treated with 10 nM DHT alone or HG122 and 10 nM DHT for 12 h. DMSO was added as the control. Total RNA was extracted using TRIzol (Takara, Japan) according to the manufacturer’s instructions. Total RNA (1 μg) was used for cDNA synthesis using a cDNA reverse transcription kit (Takara, Japan). Real-time PCR was performed in triplicates using gene-specific primers (AR-F: GGTGAGCAGAGTGCC-CTATC, AR-R: GAAGACCTTGCAGCTTCCAC; PSA-F: CTTGTAGCCTCTCGTG-GCAG, PSA-R: GACCTTCATAGCATCCGTGAG; TMPRSS2-F: CTGGTGGCTGA-TAGGGGATA, TMPRSS2-R: GGACAAGGGGTTAGGGAGAG; GAPDH-F: ACC-CAGAAGACTGTGGATGG, GAPDH-R: TTCAGCTCAGGGATGACCTT) on a Stratagene Mx3005P PCR system (Agilent Technologies, CA, USA). The mRNA expression was normalized to GAPDH expression. Data analysis was performed using the Microsoft Excel 2010 and GraphPad Prism 5 software. Data shown are representative of at least three experiments.

### Western Blotting and Immunoprecipitation

Untreated and treated cells were lysed in TNES buffer containing 50 mmol/L Tris (pH 7.5) 100 mmol/L NaCl, 2 mmol/L EDTA, 1% Nonidet P-40, and a 1 × protease inhibitor mixture (Roche Applied Science). Lysates were fractionated on polyacrylamide gels and transferred to nitrocellulose. The blots were probed with specific antibodies followed by secondary antibody then membranes were examined by the LI-COR Odyssey infrared imaging system (LI-COR Biotechnology, Lincoln NE). For the immunoprecipitation experiments, 0.5 mg of soluble cell extract was immunoprecipitated with either AR antibody and their respective IgG controls, using protein-G plus agarose (Calbiochem) as recommended by the manufacturer. Immunoprecipitated proteins and 50 mg of total cell extracts were resolved by 10% SDS-PAGE and immunoblotted for the indicated proteins. Immunoblots were analyzed using the Odyssey infrared imaging system.

### Cyclohexamide and Proteasome Degradation Assay

For cycloheximide assay, LNCaP and 22RV1 cells were treated with cycloheximide with or without HG122 for various lengths of time. AR protein level was measured by western blotting analysis. For proteasome degradation assay, LNCaP and 22RV1 cells were treated with various concentrations of HG122 with or without 10 μM MG132 and AR protein level was measured by western blotting analysis.

### *In Vivo* Tumor Growth Xenograft Model

22RV1 subcutaneous xenografts were performed, as previously described (29). BALB/c-nude mice (6 weeks old, male) were purchased from the Sino-British Sippr/BK Lab Animal Co., Ltd. (Shanghai, China). Mice were randomly divided into three groups (eight animals/group). The animal use protocol was approved by the Institutional Animal Care and Use Committee of East China Normal University. The 22RV1 xenograft tumor model was developed by injecting 3 × 10^6^ 22RV1 cell suspension into the right side of the dorsal area of a BALB/c-nude mouse using 100 μL PBS or 50% Matrigel. Tumor nodules were allowed to grow to a volume of approximately 100 mm^3^ before initiating the treatment. Tumor-bearing BALB/c-nude mice were randomly assigned to three groups and treated with the indicated compound or drug. The body weight of each mouse was recorded, and tumor volume was determined using a digital vernier caliper measurement every 3 days. At the end of the experiment, mice were sacrificed. Solid tumors were removed and processed for immunohistochemical analysis.

### Orthotopic CRPC Model

For orthotopic CRPC xenograft, male BALB/c-nude mice (8–9 weeks of age) were anesthetized using 150 mg/kg 2,2,2-tribromoethanol and 350 mg/kg tert-amyl alcohol and surgically injected with 5 × 10^5^ of 22RV1-luc cells suspended in 30 μL 50% Matrigel into the dorsolateral prostate lobes. One week after injection, the tumor-bearing mice were castrated and randomly assigned to three groups. A week later, animals were intraperitoneal injected with HG122 (10 mg/kg/d), ENZ (10 mg/kg/d), or DMSO (control). Prostate tumor growth and local metastasis were monitored weekly using the IVIS Imaging System (Xenogen Corporation, Alameda, CA, USA). Images and measurements of bioluminescent signals were acquired and analyzed using Living Image and Xenogen software.

### Histology and Immunohistochemistry (IHC)

Tumor and mouse tissue specimens were fixed in 4% formaldehyde for 12 h, processed, and embedded in paraffin blocks. Sections (4 μm) of the lungs and other tissues were stained with hematoxylin and eosin (H&E). Histopathological changes were observed under a light microscope. For immunohistochemical staining, sections were cut from the paraffin blocks and incubated with anti-Ki-67 (1:250) (Abcam, MA, USA), and anti-AR (1:50; N-20) (Santa Cruz, CA, USA) as primary antibodies. Immuno-reactivity was visualized using peroxidase-DAB22 (DAKO, Japan). Three tumors per group were analyzed. The number of Ki67-positive cells and AR-positive cells was quantified using DM 4000B photomicroscope (Leica, DE), and the apoptotic index in six random fields per group was counted.

### Statistical Analysis

Experiments were carried out in three or more replicates. Statistical analyses were performed using Student’s t-test. P-values < 0.05 were considered significant. The differences between the control group and experimental groups were determined using one-way ANOVA. Since treatment and time course were investigated, two-way ANOVA followed by *post hoc* test was also used. Data were expressed as means and 95% confidence intervals, and P < 0.05 was considered significant.

## Results

### HG122 Inhibits the Growth of PCa Cells

**Figure 1:**
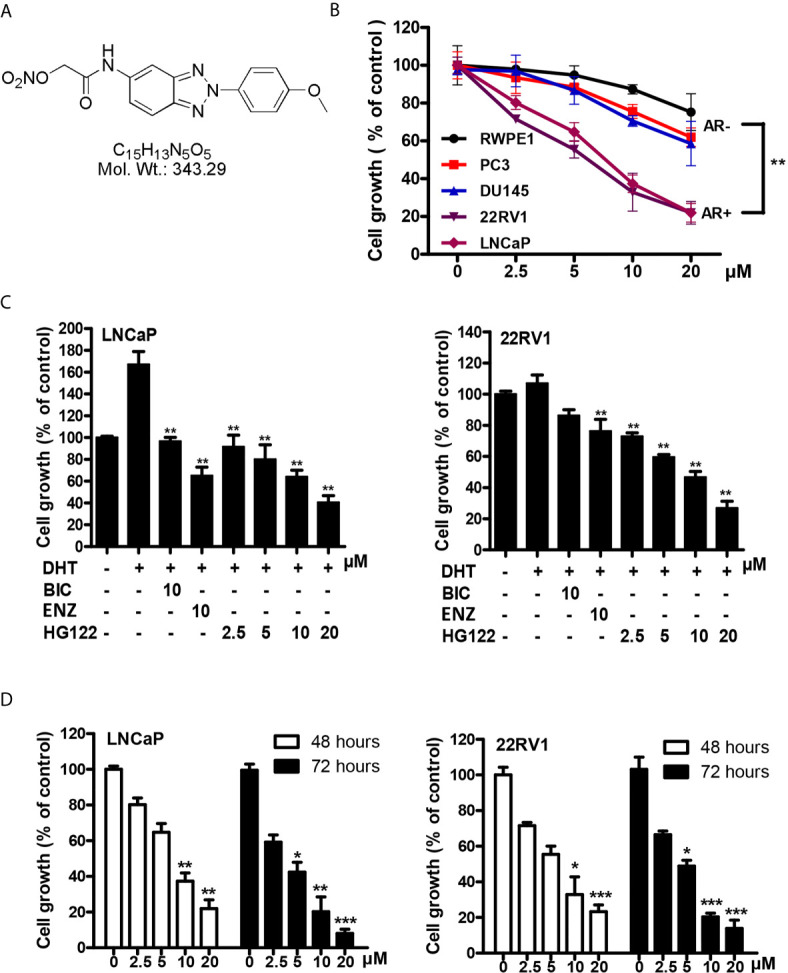
Inhibitory effects of HG122 on the proliferation of PCa cell lines. **(A)** The chemical structure of HG122. **(B)** The effect of HG122 on cell growth. LNCaP, 22Rv1, PC3, DU145, and normal prostate cells (RWPE1) were treated with HG122 for 48 h, and the viability of cells was determined using the MTS assay. **(C)** Histogram represents the effect of HG122 on DHT-induced cell growth in AR-positive cells (LNCaP and 22RV1). **(D)** The effect of HG122 on cell growth in AR-positive cells (LNCaP and 22RV1) after 48 h or 72 h. The values are represented as percent viable cells, where DMSO-treated cells are regarded as 100% viable. Each bar in the histogram represents means ± SD of three independent experiments, *, P < 0.05; **, P < 0.01; ***, P < 0.001, compared to the vehicle control group.

To identify compounds that block the transcriptional activities of AR, we used the MMTV-luciferase reporter system containing AR-binding elements (30) to screen a library of synthesized compounds available in our laboratory and identified a small-molecule compound termed HG122 (Structure in **Figure 1A**), that potently reduced the transcriptional activities of AR. Firstly, we tested the efficacy of HG122 against the growth and viability of PCa cells, including LNCaP, 22Rv1, PC3, and DU145 cell lines and normal prostate cell line RWPE1 which showed different AR statuses and functions, under standard culture conditions. PC3 and DU145 did not express AR, while LNCaP cells were androgen-dependent and expressed functional T877A-mutated AR. 22Rv1 cells were androgen-independent but responsive, expressing functional H874Y-mutated AR. Therefore, these four cell lines constituted a panel of diverse cellular models for PCa. HG122 (2.5–20 μM) treatment for 48 h resulted in a dose-dependent growth inhibition of PCa cells. LNCaP and 22RV1 cells had an IC50 of 8.7 and 7.3 μM, respectively (**Figure 1B**), while PC3 and DU145 cells had an IC50 above 20 μM. These data suggested that HG122 inhibited the growth of AR-positive cells more potently than that of AR-negative tumor cells or normal prostate cells (**Figure 1B**). Next, we investigated whether HG122 inhibited the androgen-induced growth of PCa cells. Tumor cells were tested with or without DHT. As shown in **Figure 1C**, the AR antagonists bicalutamide and enzalutamide effectively blocked cell growth, as well as HG122 in androgen-sensitive LNCaP cells. However, in the CRPC cell line 22RV1, 10 μM BIC or enzalutamide did not significantly inhibit cell growth, but 2.5 μM HG122 remarkably achieved this inhibition. Besides, HG122 inhibited AR-positive cell growth in a dose- and time- dependent manner (**Figure 1D**).

### HG122 Inhibits the Colony Formation and Migration of PCa Cells

**Figure 2:**
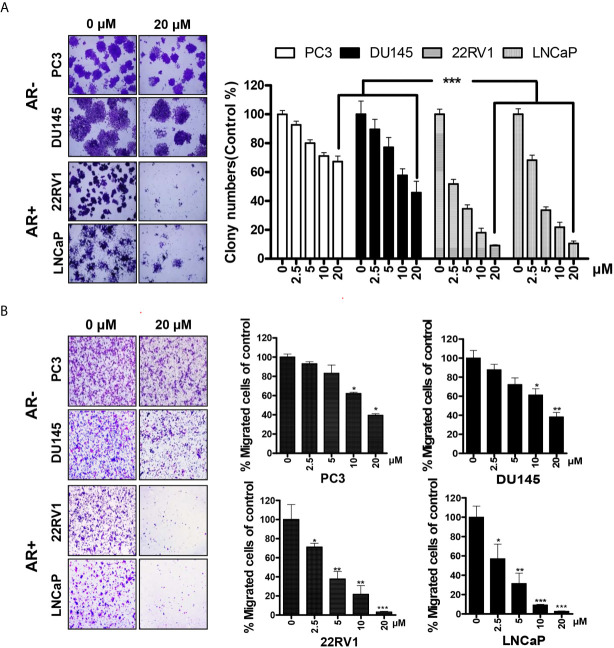
AR-positive PCa cells were more sensitive to HG122. **(A)** AR-negative cell lines PC3 and DU145 and AR-positive cell lines LNCaP and 22RV1 were treated with 0 or 20 µM HG122 in 6-well plates. After 7 days, cell colonies were counted, and colony formation for each cell line was presented (n = 3). **(B)** HG122 inhibited AR-positive cell line LNCaP and 22RV1 migration more effectively. A total of 1 × 104 PCa cells were seeded in the top chamber and treated with different concentrations of HG122, while 10 nM DHT was added in the bottom chamber. After 12 h, the cells that migrated were stained and quantified. Each bar in the histogram represents means ± SD of three independent experiments, *, P < 0.05; **, P < 0.01; ***, P < 0.001, compared to the vehicle control group.

To further explore the effects of HG122 on the growth and migration of PCa cells, colony formation assay and transwell migration assay were performed. In colony formation, AR-positive cells were more sensitive to HG122 (**Figure 2A**) and HG122 suppressed the migration of AR-positive cells more effectively than AR-negative cells in migration assay (**Figure 2B**).

### HG122 Suppresses the Transcriptional Activities of AR

**Figure 3:**
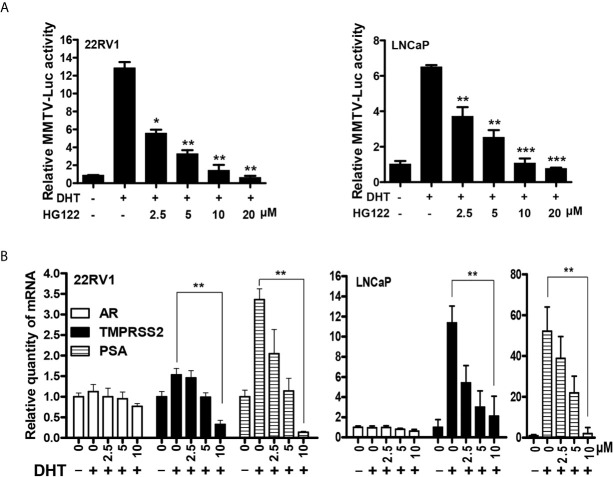
HG122 down-regulated AR target genes. **(A)** HG122 dose-dependently inhibited DHT-induced AR activity. LNCaP and 22RV1 cells were transiently transfected with MMTV-luc reporter plasmid and Renilla plasmid. Cells were treated with different concentrations of HG122 after stimulated with DHT for 12 h; the luciferase activities were measured, and results were expressed as the ratio of firefly to Renilla luciferase activity (n = 3). **(B)** HG122 down-regulated the mRNA level of PSA and TMPRSS2, but not that of AR. The mRNA levels of PSA, TMPRSS2, and AR were measured using quantitative-PCR and normalized to GAPDH. *, P < 0.05; **, P < 0.05; ***, P < 0.001.

To further test the bioactivity of HG122, luciferase reporter assays were performed in LNCaP and 22RV1 cells. HG122 inhibited the DHT-induced transcriptional activities of AR in a dose-dependent manner (**Figure 3A**). In order to examine whether HG122 affected AR-dependent endogenous gene expression, the levels of mRNA transcripts for numerous well-characterized AR-regulated genes were measured in LNCaP and 22RV1 cells. As shown in **Figure 3B**, HG122 decreased the mRNA expression of PSA and TMPRSS2 in LNCaP and 22RV1 cells.

### Induction of AR Degradation Through the Proteasome-Mediated Pathway by HG122

**Figure 4:**
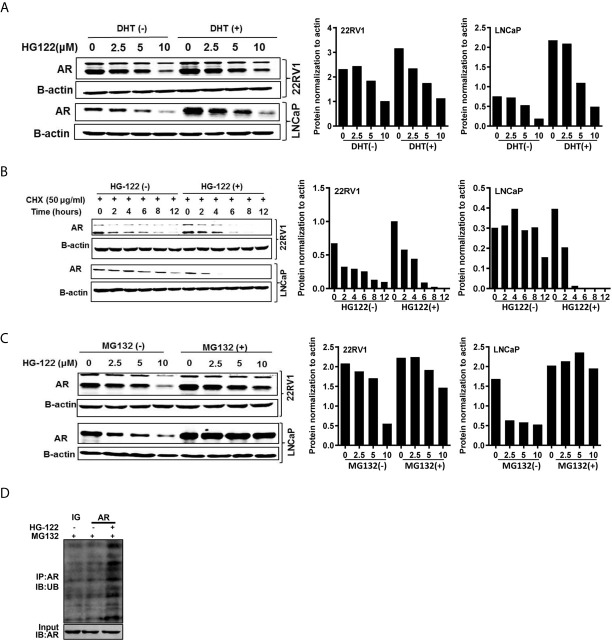
HG122 degraded AR protein. **(A)** HG122 down-regulated AR protein. LNCaP and 22RV1 cells were treated for 12 h with indicated concentrations of HG122 with or without DHT, and the AR protein level was measured using western blotting. **(B)** AR protein half-life was shortened by HG122. LNCaP and 22RV1 cells were treated with cycloheximide with or without HG122 for various periods. The AR protein level was measured using western blotting. **(C)** The HG122-induced decrease in AR protein was rescued by the proteasome inhibitor MG132. LNCaP and 22RV1 cells were treated with various concentrations of HG122 with or without 10 μM MG132, and the AR protein level was measured using western blotting. **(D)** HG122 induces ubiquitination of AR. LNCaP cells were treated with or without HG122 in the presence of MG132. Immunoprecipitation (IP) was done using anti-AR and immunoblotting was done with anti-ubiquitin antibody.

To investigate the mechanism of AR transcriptional activity inhibition by HG122, we first determined the AR protein level after HG122 treatment in PCa cells. HG122 potently reduced AR protein expression in a dose-dependent manner in LNCaP and 22RV1 cells (**Figure 4A**). We observed that HG122 reduced the AR protein level, not only in the absence of DHT, but also in the presence of DHT in AR-positive PCa cells, in which AR was more stable and had a higher basal level in the presence of the ligand. Notably, HG122 down-regulated the truncated splice variants of AR that were continually activated and resistant to AR antagonist therapy in 22RV1 cells. To investigate why HG122 reduced the expression of AR, we tested the effect of HG122 on AR protein stability. Surprisingly, AR protein stability was significantly reduced by HG122 (**Figure 4B**). To test whether HG122 induced AR degradation through the proteasome pathway, we treated cells with the proteasome inhibitor MG-132, which resulted in a marked suppression of HG122-induced AR depletion (**Figure 4C**). Further study also confirmed that HG122 induced the ubiquitination of AR (**Figure 4D**). These data suggested that HG122 induced AR degradation through the proteasome-mediated pathway.

### HG122 Blocks Tumor Growth on the 22RV1 Subdermal Model

**Figure 5:**
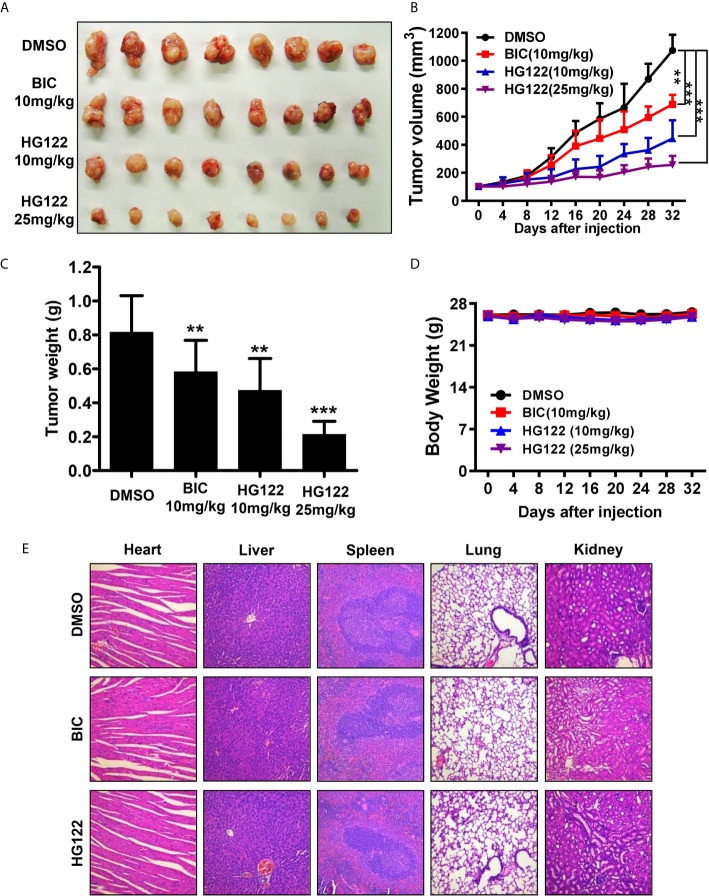
Therapeutic effects of HG122 on subdermal model. **(A)** 22RV1 tumor-bearing nude mice were treated with HG122 at 10 and 25 mg/kg/d, BIC at 10 mg/kg/d or with vehicle. Compared to the vehicle group, HG122- and BIC-treated group showed inhibition of tumor growth. **(B)** Tumor volumes were measured twice per week, and treatment with HG122 resulted in significant inhibition of tumor volume compared to treatment with the vehicle control. **(C)** Tumor weights were measured. **(D)** HG122 did not cause obvious changes in body weight in the vehicle and HG122-treated groups. **(E)** HG122 (25 mg/kg/d) did not cause obvious pathological abnormalities in normal tissues. H&E staining of paraffin-embedded sections of the liver, spleen, kidney, heart, and lung (n = 8, **, P < 0.01; ***, P < 0.001).

To determine the anti-androgen and anti-tumor activities of HG122 *in vivo*, 22RV1 xenograft CRPC model was used. As shown in **Figures 5A–C**, 22RV1 xenografts were resistant to 10 mg/kg/d BIC administration with only 30% tumor growth inhibition, while administration of HG122 significantly inhibited the tumor growth in 22RV1 xenografts. The tumor inhibition rate of 10 mg/kg/d HG122 treatment group reached 60%, and even closer to 80% at the dose of 25 mg/kg/d. Importantly, no significant differences in body weights were found among the HG122 treatment groups and DMSO control group (**Figure 5D**). Similarly, no significant differences in the daily consumption of diet and drinking water of the mice in the three groups and no physical sign of toxicity were observed in the HG122-treated group. Furthermore, toxic pathologic changes in the hearts, livers, spleens, lungs, and kidneys were not detected by H&E staining (**Figure 5E**). These data suggested that the inhibition of tumor growth was not attributable to systemic toxicity.

### HG122 Blocks Tumor Growth and Metastasis on Orthotopic Castration-Resistant Xenograft Model

**Figure 6:**
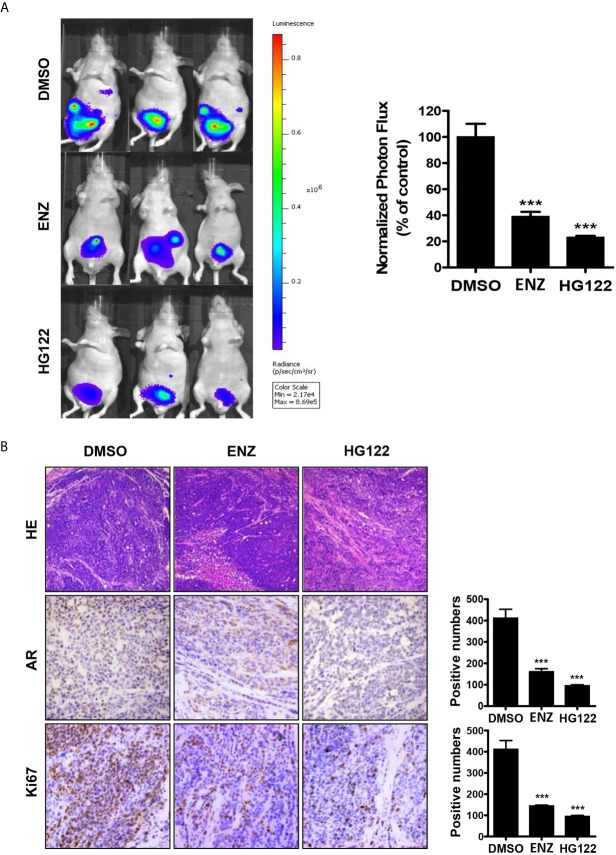
Therapeutic effects of HG122 on orthotopic castration-resistant xenograft model. **(A)** Bioluminescent analyses of orthotopic prostate tumors treated with HG122, ENZ, or vehicle control. Tumors were imaged every week to observe local tumor growth and evidence of tumor cell dissemination. Representative images of three mice per group are illustrated (n = 5). ***P < 0.001. **(B)** Ki67, AR immunohistochemistry, and the structural changes in tumor were analyzed. Photographs of representative tumor sections were taken at ×200 magnification (n = 4; ***P < 0.001).

An orthotopic xenograft model was used to evaluate the effect of HG122 on both tumor growth and metastasis. As shown in **Figure 6A**, 22RV1 orthotopic xenograft tumor growth in castrated mice was also regressed by administration of HG122, while these CRPC xenografts were partially resistant to enzalutamide treatment. Although both enzalutamide and HG122 basically inhibit the metastasis of prostate cancer, but HG122 obviously has the more obvious inhibition effect on the growth of orthotopic prostate tumor. HG122 (10 mg/kg/d) administration reduced the tumor volume by 82%, while enzalutamide administration reduced the tumor volume only by 60%. The tumor histological changes in mice bearing 22RV1 xenograft were observed using light microscopy after H&E staining. As shown in **Figure 6B**, an ap-parent decrease in expression of AR and Ki67 proteins was detected in cells in HG122-treated xenografts compared to the control group (**Figure 6B**).

## Discussion

PCa remains a major cause of cancer-related morbidity globally, and the majority of PCa-related deaths result from castrate-resistant disease (31). Androgen deprivation has been used as a standard therapy for treating PCa in humans. However, using anti-androgens to treat PCa of all types is limited (32, 33). Reports suggest that anti-androgen agents decrease tumor androgen concentrations and cause death of normal prostate epithelial cells, exposing surviving PCa cells to a relative abundance of androgen (2, 3, 34).

Agents that exhibit the potential to combat AR under both androgen containing and non-androgen-containing environments are rare but desired. The results of this study are important because they show that HG122 inhibits the growth and proliferation of AR-positive PCa cells presenting different androgen sensitivity and AR expression status. HG122 inhibited proliferation and invasion of PCa cells in a concentration-dependent manner, and have little effect on the endothelial cells. Moreover, administration of HG122 had excellent efficiency for blocking the growth of CRPC xenograft tumors.

AR, the primary regulator for PSA expression (35), which induces PSA expression through AREs containing enhancer elements located in the proximal 6 kb of the PSA promoter (36). PSA is also found in PCa cells that express AR but are nonresponsive to androgen (37). HG122 potently blocked the activities of AR with a lower level of PSA and TMPRSS2. The observations that HG122 treatment reduced PSA levels in CRPC cells have high clinical significance. Notably, HG122 potently degraded AR protein through the proteasome-mediated pathway. Since AR is a critical factor in CRPC, and a low AR protein level means less activation of AR signal pathway.

Recent reviews suggested that a significant proportion of CRPC remained depending on AR signaling (5, 38). Given the importance of AR for both androgen-dependent PCa (ADPC) and CRPC, our data provide evidence that HG122 is a novel agent that can disrupt AR signaling in CRPC cells. Collectively, these data shown HG122 inhibited AR signaling in PCa cells and animal models irrespective of the AR and androgen sensitive status by adopting several approaches, which implied HG122 as a more effective disruptor of AR signaling comparing to known anti-androgens (e.g., BIC). We, thus, suggest that HG122 might be a potential candidate for cancer prevention.

Of course, we also noticed that HG122 not only had a significant inhibitory effect on androgen receptor-positive prostate cancer cells through cell proliferation, clone formation and cell migration assays which mainly related to the degradation of androgen receptor, AR-negative PC3 and DU145 cells also showed a dose-dependent inhibition of HG122 in colony formation and cell migration. We believed that HG122 had a mechanism to inhibit cell function beyond AR signal pathway which need further study in the future.

Overall, this study showed that HG122 potently inhibited the tumorigenicity of 22Rv1 and LNCaP cells, which were androgen-independent but sensitive under *in vivo* conditions. HG122 decreased the protein level of AR and the expression of AR target genes, resulting in cell growth inhibition and metastasis suppression of CRPC. We report here the mechanism-based anti-AR activity of HG122 in CRPC cells under *in vitro* and *in vivo* conditions. We suggest that HG122 is a potent inhibitor of AR and could be used as a therapeutic agent to treat CRPC with considerable clinical relevance.

## Data Availability Statement

The original contributions presented in the study are included in the article/**Supplementary Material**, further inquiries can be directed to the corresponding authors.

## Ethics Statement

The animal study was reviewed and approved by Animal Investigation Committee of the Institute of Biomedical Sciences, East China Normal University.

## Authors Contributions

Conceptualization, SP and TD. Methodology, XC, YH. Chemical synthesis, HW and DW. Validation, XC, JZ, and YL. Investigation, XC and TS. Data curation, XC, YH TS, and YL. Writing—original draft preparation, XC and YH. Writing—review and editing, ZY, SP, and TD. Visualization, XC and SP. Supervision, XC, YH and TD. Project administration, SP and TD. Funding acquisition, ML, ZY, SP, and TD. All authors contributed to the article and approved the submitted version.

## Funding

This study was supported by National Key R&D Program of China (2018YFA0507001 to ML), National Natural Science Foundation of China (81830083 to ML; 82073310 and 81773204 to ZY; 81802970 to SP), Innovation program of Shanghai municipal education commission (2017-01-07-00-05-E00011 to ML), the Fundamental Research Funds for the Central Universities (SP), China Postdoctoral Science Foundation (2018M632065 to SP), The Science and Technology Commission of Shanghai Municipality (11DZ2260300 to ML and 20JC1417900 to ZY), Shenzhen Municipal Government of China (KQTD20170810160226082 to ML), Science and Technology Commission Fund of Shanghai Fengxian District (20160907 to TD), ECNU Construction Fund of Innovation and Entrepreneurship Laboratory (44400-20201-532300/021 to ZY) and ECNU Public Platform for innovation (011).

## Conflict of Interest

The authors declare that the research was conducted in the absence of any commercial or financial relationships that could be construed as a potential conflict of interest.
