# Supplementary figures and images for: Regression of Castration-Resistant Prostate Cancer by a Novel Compound HG122
Source: Front Oncol. 2021 Jun 3;11:650919. doi: 10.3389/fonc.2021.650919 (PMC8210671; doi:10.3389/fonc.2021.650919)

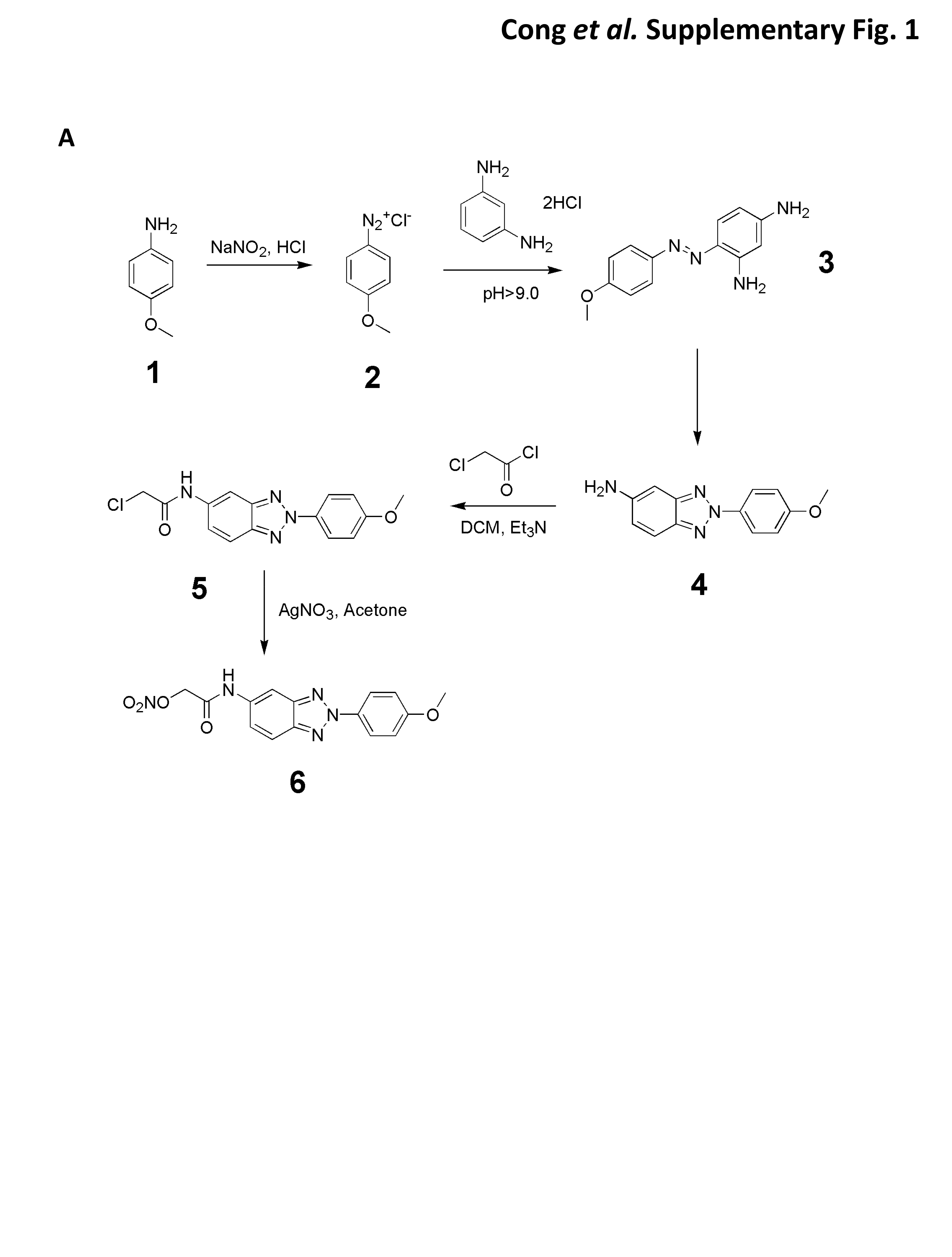

Supplement: Supplementary file 1 [file Image_1.jpeg]
